# The spectrum of pathological findings in coronavirus disease (COVID-19) and the pathogenesis of SARS-CoV-2

**DOI:** 10.1186/s13000-020-00999-9

**Published:** 2020-07-14

**Authors:** Rolf F. Barth, L. Maximillian Buja, Anil V. Parwani

**Affiliations:** 1 Department of Pathology, The Ohio State University, Columbus, OH 43210 USA; 2 Department of Pathology and Laboratory Medicine, University of Texas Health Science Center, Houston, TX 77030 USA

The coronavirus disease (COVID-19) is now a worldwide pandemic and is the most significant global health crisis of our time. COVID-19 disease continues to present us with major healthcare challenges with over 8 million cases around the world and still less than optimal treatment options [1]. The virus initially was identified in Wuhan, Hubei Province, China in 2019. COVID-19 disease is caused by a novel coronavirus, which has been named “Severe Acute Respiratory Syndrome Corona virus-2 (SARS-CoV-2)” [2]. COVID-19 is highly transmissible among humans and primarily, but not only, affects the lower respiratory tract and lungs. The virus attaches to the angiotensin converting enzyme-2 (ACE-2) receptor in order to help enter the target cell by attaching its spike-like surface projections (“corona”) to the receptor. This receptor is expressed on various cell types such as myocardial cells, type II pneumocytes, enterocytes and vascular endothelium. Most of the symptomatic patients have mild flu-like features but a significant subset develop a bronchopneumonia, which clinically is the acute respiratory distress syndrome (ARDS), and leads to significant morbidity and mortality [3]. The fatality rates of COVID-19 are highest amongst older patients with concomitant comorbidities and/or patients who are immunosuppressed [4]. Our current understanding of the pathology and the pathogenesis of COVID-19 disease and SARS-CoV-2 transmission is at an early stage and much still remains to be learned [5, 6]. Additionally, there are also published reports on the pathogenesis of other coronaviruses such as SARS-CoV and MERS-CoV [7–9].

In early April a Letter to the Editor appeared online in Chest entitled “A call to action: the need for autopsies to determine the full extent of organ involvement associated with COVID-19” [10]. This was prompted by the stunning lack of publications relating to autopsy findings in decedents who had succumbed to COVID-19 infections despite the fact that several hundred thousand individuals already had died by that time [11]. Since then, at the time of this writing in late June and to the best of our knowledge, there have been at least 13 reports describing autopsy findings in approximately 250 decedents who have succumbed to COVID-19 [4, 12–22] In the meantime, as of June 15, 2020, the number of confirmed cases has climbed to over 2.1 million and over 115,000 deaths in the United States and 8 million cases and more than 440,000 deaths worldwide. Therefore, the total number of autopsies performed is miniscule compared to the number of deaths, but nevertheless they are both very revealing and important in order to better understand the multi-organ involvement associated with COVID-19 infection and for the development of better treatment strategies [1, 3].

The three largest and most recent of these are reports from the Mount Sinai Hospital in New York City of 67 decedents [12], the L. Sacco Hospital in Milan, Italy of 38, limited to the lungs [14] and the University Hospital in Basel Switzerland of 21 decedents [19]. These three reports taken together have provided a more complete picture of the various organs that can be involved in individuals who succumb to COVID-19. What initially was thought to be a disease almost exclusively involving the lungs now is being recognized as one that involves multiple organ systems including the heart, kidneys, bone marrow, lymph nodes and brain. The underlying pathology of COVID-19 infection appears to be involvement of vascular endothelium of multiple organs [4].

It is noteworthy that in the Chinese medical literature there was, as of March 2020, only one autopsy report of a decedent who had succumbed to COVID-19 and this was published in Chinese in the Journal of Forensic Medicine [23]. Unfortunately, no diagnoses were given nor was microscopic examination carried out making this report of very limited usefulness. This in part is due to the fact that very few autopsies are carried out in China and most of these seem to be forensic autopsies. Several other reports from China have provided some detailed histopathologic information based on “biopsies” of the lungs and kidneys of a few decedents taken following their death [8, 24, 25]. Even with this limitation, very important information was obtained, which described the major hallmark of COVID-19 infection, namely diffuse alveolar damage (DAD) of the lungs and a glomerulopathy involving the kidneys. Although autopsies have shown a steep decline in almost all countries of the world, they still remain a very powerful tool [26] to develop a full understanding of a new and previously unknown diseases such as SARS-CoV2. It is beyond the scope of this Editorial to go into a detailed discussion of the specific pathologies associated with COVID-19 infection, but, based on the autopsy reports to date, a much clearer picture is emerging and will be briefly summarized here. Readers interested in more detailed information are referred to the report cited in the references in this editorial. For a more comprehensive overview of SARS-CoV2 and COVID-19 disease readers are referred to a soon to be published review by Mohanty et al., 2020 [27].

Acute severe COVID-19 respiratory disease develops as a severe acute respiratory distress syndrome (ARDS) that autopsy studies have shown to be related to an underlying severe form of DAD in the acute, exudative phase. COVID-19-induced DAD is characterized by damage to alveolar capillary endothelium and type II pneumocytes leading to alveolar septal edema and the formation of hyaline membranes, accumulation of numerous megakaryocytes, platelets, and neutrophils in alveolar capillaries and precipitation of fibrin inside and outside the alveolar capillaries with a relatively mild accumulation of lymphocytes and macrophages within alveoli. The former provides evidence for a pulmonary thrombotic microangiopathy that often results in fibrin-platelet thrombi in alveolar capillaries and small pulmonary arteries. Similar changes have been identified in DAD of other etiologies, including influenza and SARS-CoV [7]. However, the changes in full blown COVID-19 DAD are more extensive and severe, and collectively constitute a distinct and characteristic type of COVID-19 DAD [13]. The pulmonary thrombotic microangiopathy progresses to a diffuse hypercoagulable state in some patients that can lead to deep vein thrombosis and large pulmonary thromboemboli. A clinical marker for patients at risk for this coagulopathy is elevated plasma D-dimer at the time of presentation. A postulated underlying mechanism for severe COVID-19 associated pneumonia is a state of virally-induced hyper-inflammation that has been variously designated as macrophage activation syndrome (MAS), cytokine storm and secondary hemophagocytic lymphohistocytosis (sHLH) [28, 29]. This hyper-inflammatory response most likely involves activation of the innate and acquired immune systems [28, 29] Hence, the initial pulmonary pathology is a florid DAD with an immuno-thrombotic microangiopathy [4, 28, 29]. Patients who succumb after a more prolonged clinical course are likely to show late stage DAD and/or organizing pneumonia. Consensus guidelines are now available for prevention, antithrombotic therapy, and follow-up for thrombotic and thromboembolic disease in COVID-19 patients [30] and the damping of the hyperimmune inflammatory response by the administration of Dexamethasone [31].

The Chinese clinical studies early on identified serum troponin as a marker for an adverse outcome and this led to the suggestion of a myocarditis [24, 25]. However, cardiac involvement has proven to be more subtle with most hearts showing multifocal individual cardiomyocyte injury without overt interstitial inflammation and only a few cases of classical myocarditis have been described [13]. The pathological basis for severe acute renal failure in some patients requires further evaluation. Some decedents had prominent thrombi in glomerular capillaries [19] although in many cases, glomerular involvement has been limited. It is likely that the acute renal failure is a secondary form of acute tubular necrosis [19]. The spleen has been found to have a dimunition of white pulp with loss of peripheral cuff lymphocytes. This is consistent with a viral attack on immunocytes and the lymphopenia that may be seen at the time of presentation. Hemophagocytic lymphohistiocytosis (HLH) has been postulated to have an important role in the progression of severe COVID-19 disease [28, 29]. However, so far morphological evidence of prominent erythrophagocytosis has been found in only a few cases [12].

Of special interest is involvement of the brain in individuals infected by COVID-19. There certainly was a hint of this in the very common presenting symptoms of anosmia and ageusia suggesting the neurotropism of the virus. Magnetic resonance imaging of the brain of one patient with these two symptoms revealed abnormalities in the right gyrus rectus and the olfactory bulbs [32]. Even more striking has been numerous reports of individuals with COVID-19 infection who have had strokes, and in some instances these were the presenting symptom. Bryce et al. have reported that 6 of 20 brains that were examined showed a range of abnormal pathologic findings, the most striking of which was the presence of microthrombi and acute infarcts in the brains that were examined. In some brains this was accompanied by acute parenchymal microthemorrhages in areas of necrotic infarcts, which may be due to the high expression of ACE2 in vascular endothelium, not only in the brain [33] but also in the lungs. In contrast to these findings, Solomon et al. [34] have reported that neuropathologic examination of the brains of 18 decedents failed to reveal any changes that could specifically be related to COVID-19 infection. However, if COVID-19 is anything like SARS-CoV [7], then the possibility of neuronal infection also must be considered. More detailed neuropathologic examination of a larger number of decedents with a broader representation in age, gender, and duration of clinical course should provide a more definitive answer to the question of brain involvement.

Finally, the involvement of other organ sites such as the testes, gastro-intestinal tract, liver, endocrine system and musculo-skeletal system are yet to be determined. We still are at an early stage of understanding the effects of COVID-19 on the immune system but clearly this is of great importance considering the occurrence of a hyperactive immune response to the virus [28, 29]. The autopsy reports that already have been published provide a solid base for a better understanding of the consequences of COVID-19 infection but much more remains to be learned about this complex disease in order to develop better treatment strategies. This includes a better understanding of the epidemiology, pathology, molecular profile and immunology of the virus and the need for better diagnostic tests including serological assays to monitor the host response to the virus, recovery and the persistence of disease in infected individuals. All of these would help in the design of better therapeutics and vaccines [35, 36].

In closing, this editorial announces the inauguration of a new thematic series on the pathological spectrum and pathogenesis of SARS-CoV-2. As the editor-in-chief of Diagnostic Pathology, I, Anil Parwani, am very excited about launching this important series as it will serve to attract key articles in COVID-19 pathophysiology, diagnostics, molecular pathology, immunology with an emphasis on the clinio-pathological correlation of this disease. The series will also solicit articles on the most up to date understanding of the histopathological features of a SARS-CoV-2 infection including an understanding of the ultrastructure of the virus. We hope that our readers will find the collection of articles in this series both interesting and useful in disseminating important information relating to the pathology of COVID-19.
